# Growth and Eating Behaviours at 2 Years Corrected Age in Extremely Low-Birthweight Babies; Secondary Cohort Analysis from the ProVIDe Trial

**DOI:** 10.3390/nu16234095

**Published:** 2024-11-27

**Authors:** Morgan J. Easton, Frank H. Bloomfield, Yannan Jiang, Barbara E. Cormack

**Affiliations:** 1 Liggins Institute, University of Auckland, Auckland 1023, New Zealand; measton@adhb.govt.nz (M.J.E.); f.bloomfield@auckland.ac.nz (F.H.B.); y.jiang@auckland.ac.nz (Y.J.); 2 Starship Child Health, Auckland City Hospital, Auckland 1023, New Zealand; 3 Department of Statistics, University of Auckland, Auckland 1010, New Zealand

**Keywords:** preterm, nutrition, neonatal, premature, newborn, gestational age, conditional growth, body composition, body mass

## Abstract

Early postnatal growth following extremely preterm birth may have long-term effects on growth, eating behaviours and health. Background/Objectives: To determine whether growth to age two years is conditional on growth in the NICU, a conditional growth analysis was performed in a cohort of 330 extremely low-birthweight (ELBW; birthweight < 1000 g) participants in the ProVIDe trial who were followed-up at 2 years corrected age (CA); Methods: We used z-score change for weight, length and head circumference from 36 weeks post-menstrual age to 2 years CA as the end-point-adjusted for birth z-score and z-score change from birth to 36 weeks. Growth and body composition were assessed using bioimpedance analysis. Relationships between eating behaviours and body mass index (BMI) at 2 years CA and growth were assessed using a Child Eating Behaviour Questionnaire (CEBQ) completed by parents at 2 years CA; Results: Growth, or change in z-score, from 36 weeks PMA was conditional upon growth in the NICU, with slower neonatal growth associated with faster early childhood growth (weight: R^2^ = 0.27, ß-coefficient −0.81 (95% CI: −0.96, −0.66), *p* < 0.0001; length: R^2^ = 0.28, ß-coefficient −0.64 (95% CI: −0.76, −0.51), *p* < 0.0001; head circumference: R^2^ = 0.18, ß-coefficient −0.61 (95% CI: −0.76, −0.46), *p* < 0.0001). Fat-free mass index, adjusted for confounding factors, was positively correlated with z-score change from NICU discharge to 2 years CA for weight, but not length (weight: R^2^ = 0.50, ß-coefficient = 0.87 (95% CI: 0.56, 1.18), *p* < 0.0001; length: R^2^ = 0.32, ß-coefficient = 0.01 (95% CI: −0.40, 0.42), *p* = 0.95). At 2 years CA, CEBQ scores for enjoyment were significantly higher and satiety and slowness significantly lower in children with a BMI ≥ 90th percentile than in children with a BMI ≤ 10th percentile or between the 10th−90th percentile.; Conclusions: Growth from NICU discharge to 2 years CA is conditional upon growth in the NICU, with slower NICU growth linked to faster early childhood growth, and weight z-score changes positively correlated with fat-free mass index. At age 2, children with a BMI ≥ 90th percentile have significantly different eating behaviour assessments by caregivers compared to children with a BMI ≤ 10th percentile or between the 10th–90th percentile; further RCTs are needed to confirm links between nutrition factors and growth outcomes in ELBW infants.

## 1. Introduction

Extremely low-birthweight (ELBW) babies are usually the most preterm newborns, often with a gestational age of <28 weeks at birth. Their intrinsic immaturity affects all organ systems, which may have lifelong consequences for their growth and development [1]. The American Academy of Paediatrics (AAP) and the European Society for Paediatric Gastroenterology Hepatology and Nutrition recommend that preterm babies should grow similarly to a normal fetus of the same postmenstrual age, coupled with acceptable functional development [2,3]. Thus, after allowing for early postnatal fluid loss, the aim is growth approximately parallel to growth chart curves for weight, length and head circumference (HC) and to prevent large deviations from expected patterns, i.e., faltering growth or accelerated growth [4]. Faltering growth is defined as a fall in z-score for a weight of ≥1.0 that occurs over a period of 1 month or more, not including the first 2 weeks after birth [5]. Accelerated growth is an upward crossing of centiles in weight that does not come after a period of growth faltering [5]. Many ELBW babies grow slowly after birth, followed by catch-up growth during their first years [6]. Catch-up growth refers to a physiological increase in growth velocity and subsequent increase in z-score for weight after a period of faltering growth, ideally to original or birth z-score for weight. Avoiding faltering growth may be important for optimal neurodevelopmental outcomes [7], but rapid growth or weight gain with upwards crossing of percentiles in preterm babies during the early postnatal period may increase the risk of obesity and metabolic syndrome in later life [8,9,10].

Post-discharge nutrition and growth, especially in the first two years after birth, can influence health outcomes during childhood and through to adulthood [11]. However, deciding how to feed a preterm baby after hospital discharge can be difficult. Both families and healthcare professionals strive to achieve ‘normal feeding’ as quickly as possible. This can be challenging, as children born preterm are more likely to develop problematic feeding behaviours compared to those born at term [12]. In preterm-born toddlers between the ages of 12–23 months, problematic feeding, defined as the ‘child being unable or unwilling to safely eat and/or drink enough to obtain appropriate nutrition and hydration’, may be as high as 33% [13]. Despite this, there is little consensus on nutrition guidelines during neonatal intensive care unit (NICU) or on post-discharge feeding practices to support optimal growth and long-term health outcomes for ELBW babies.

The ProVIDe trial randomised 434 ELBW babies admitted to six New Zealand and two Australian neonatal intensive care units (NICUs) to receive to either 1 g·d^−1^ amino acid or placebo in addition to usual parenteral nutrition for the first 5 days after birth [14,15]. The aim of this trial was to assess the effect of an extra 1 g·d^−1^ of parenteral protein in the first five days after birth on neurodevelopment outcomes at 2 years corrected age (CA). The primary outcome was survival free from neurodisability assessed using Bayley III and neurological examination at 2 years CA. The trial showed no difference in growth at 2 years CA between the intervention and placebo groups. Our study is a secondary cohort analysis of the ProVIDe trial participants at 2 years CA, carried out to investigate relationships between growth from birth to 36 weeks post-menstrual age (PMA) and subsequent eating behaviours, growth and body composition at 2 years CA.

## 2. Materials and Methods

### 2.1. Study Population

The ProVIDe trial (Australian New Zealand Clinical Trials Registry; ACTRN12612001084875) was a multicentre, two-arm, double-blind, parallel, randomised, controlled trial conducted in 6 New Zealand and 2 Australian NICUs [14,15]. Babies were eligible if they had a birthweight < 1000 g and an umbilical arterial catheter in situ in an acceptable position. Babies were recruited between 29 April 2014 and 30 October 2018. Exclusion criteria were as follows: admission to NICU > 24 h after birth; multiple births of greater than two babies; known chromosomal or genetic abnormality; congenital disorder affecting growth; inborn error of metabolism; or in danger of imminent death. Eligible babies were randomised to the intervention or placebo group within 24 h after birth. De-identified prospectively collected demographic, growth and nutrition intake data during neonatal care were downloaded to a Microsoft Excel spreadsheet and analysed using the Statistical Discovery software JMP^®^ 12.

### 2.2. Growth Assessments

Anthropometric measurements of weight, length and head circumference at birth and subsequent measurements at 36 weeks PMA (±10 days) and at 2 years CA were taken by trained staff to ensure standardisation and accuracy. For measurements taken from birth to discharge, weight was measured using electronic scales accurate to ±10 g while infants were naked, length was measured from crown-to-heel using a neonatometer when clinically feasible or a non-stretch tape measure and head circumference was measured using a non-stretch Teflon head tape. For anthropometric measurements at the 2-year follow-up, weight was measured on a paediatric digital scale, height was measured using a stadiometer and head circumference was measured using a non-stretch tape measure. If the child was unable to stand, length was measured using a standardised length board. For the children where only length was measured, 0.7 cm was subtracted to convert it to height [16]. The Fenton dataset was used to calculate z-scores for each measurement up to 50 weeks gestational age [17]. As there are currently no birth length and head circumference data for babies born < 23 weeks gestational age in the Fenton dataset, the INTERGROWTH fetal ultrasound data on head growth [18] was examined and relatively linear growth was determined through 20 to 24 weeks gestational age. The rate of change in the Fenton length data between 23 and 28 weeks was examined and found to be slower at lower ages. Thus, to extrapolate head and length measures to 22 weeks, the trends in linear head growth and decreasing rates of length change were continued. Smallness for gestational age assessment was based on the same Fenton dataset. Longitudinal growth was assessed by calculating z-score change over time, between birth and 36 weeks PMA and between 36 weeks PMA and 2 years CA [19].

Body composition was measured at 2 years CA by bioelectric impedance analysis (BIA) (ImpediMed Imp SFB7, MediMark Europe Sarl, Grenoble, France). Prior to the measurement, height and weight were measured to the nearest 0.5 cm and 0.1 Kg, respectively. Electrodes were placed on the right hand and foot. The child’s details were entered into the machine: sex, weight, height and age. Two measurements were recorded whilst the child’s limbs were not crossed, with legs apart, and the child was not in direct contact with another person or with any metal surface. If there was a difference greater than 1% in percentage fat between the two measurements, a third measure was taken. Fat-free mass index was calculated as follows: fat-free mass (Kg) ÷ (length or height)^2^ (m^2^). Body mass index was calculated as follows: weight (Kg) ÷ (length or height)^2^ (m^2^).

### 2.3. Follow-Up at 2 Years

Parents of babies enrolled in the ProVIDe trial gave consent for follow-up at two years CA at the time of recruitment to the study. The families for whom contact information was available were sent an invitation to participate in the ProVIDe 2-year follow-up study when the child was nearing 2 years CA. Families with no current contact information available were traced via their primary health provider, grandparents or other family members. Families who agreed to take part in the follow-up were either posted questionnaires as one booklet prior to the follow-up visit or completed the questionnaire during the child’s neurodevelopment assessment. The questionnaire booklet included the ProVIDe Home and Family Questionnaire and the Children’s Eating Behaviour Questionnaire [20]. Socioeconomic status was based on the New Zealand Deprivation Index 2018 using the child’s address and grouped into quintiles ranging from 1 (least deprived) to 5 (most deprived) [21].

### 2.4. Analysis and Statistical Methods

Descriptive summaries are presented using the mean (standard deviation [SD]) or median (interquartile range [IQR]) as appropriate for continuous variables and as a number (%) for categorical variables. Two-sample *t*-tests were used to compare the means of continuous variables between groups. Linear regression models were used to evaluate the association with continuous outcome variables and are presented using the ß-coefficient (standard error [SE]; or 95% confidence interval [CI]). Tukey’s HSD test was used to compare the means between categorical independent variables with three or more categories. Pearson’s chi-square test was used to determine whether the distribution of categorical variables was different from expectations. Statistical tests were two-sided at a 5% level of significance. Conditional growth was modelled using Model 2 and 3 from Johnson et al. and adjusted for confounding variables [22]. Conditional growth analysis for size used z-score change from 36 weeks PMA to 2 years CA as the end point and included birth z-score and z-score change from birth to 36 weeks PMA as variables, as per Model 2; for body composition at 2 years CA, z-score change from birth to 36 weeks PMA and z-score change from 36 weeks PMA to 2 years CA were the variables, as per Model 3 [22]. In the conditional growth model, we adjusted for sex, SGA, index of deprivation quintiles and child’s ethnicity.

### 2.5. Ethical Approval

Ethical approval was obtained from the Northern B Health and Disability Ethics Committee (13/NTB/84, date: 11 July 2013), and each participating site had institutional approval through local institutional review processes.

## 3. Results

**Figure 1:**
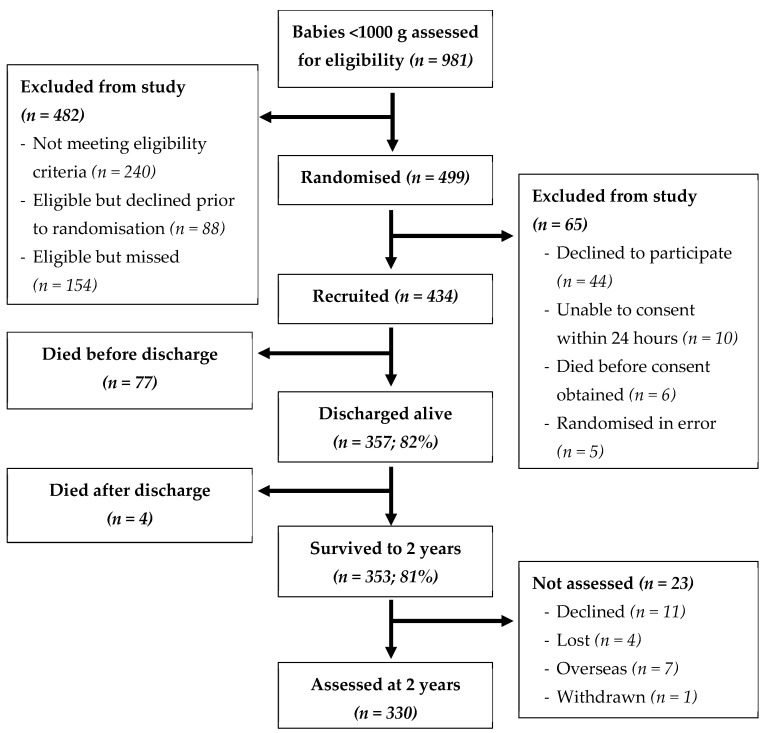
Consort flow diagram.

Of the 434 ProVIDe trial recruits, 330 were followed-up at 2 years CA, corresponding to 76% of the total recruited, and 94% of those were alive at 2 years, as shown in Figure 1.

Baseline characteristics of the recruited babies are shown in Table 1.

**Figure 2:**
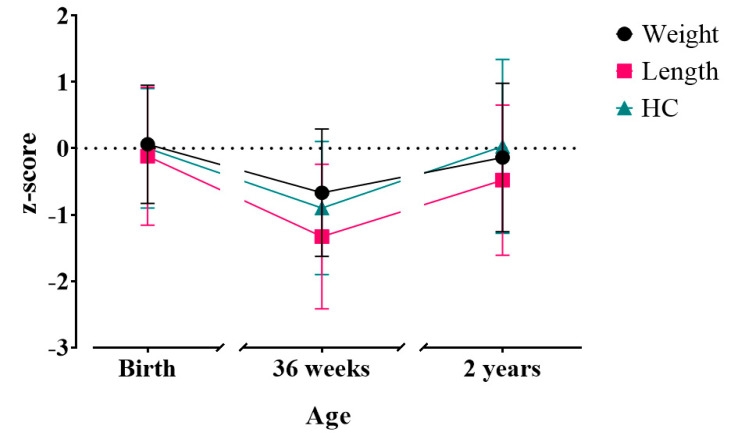
Mean z-score for weight, length and HC at birth, 36 weeks post-menstrual age and 2 years corrected age. (Error bars represent standard deviation). Weight: at birth, *n* = 434; at 36 weeks, *n* = 362; at 2 years, *n* = 321. Length: at birth, *n* = 413; at 36 weeks, *n* = 350; at 2 years, *n* = 312. HC, head circumference: at birth, *n* = 431; at 36 weeks, *n* = 356; at 2 years, *n* = 305. Data at birth and 36 weeks post-menstrual age presented previously [23].

At birth, mean weight, length and head circumference z-scores were close to zero; however, by 36 weeks PMA, weight had fallen by 0.6 and length and head circumference by 1.2 and 0.9 z-scores, respectively [23]. At 2 years CA, weight and length z-scores at 2 years CA both increased from the value at 36 weeks PMA but remained lower than the birth z-scores. Head circumference z-score mean returned to near the mean birth z-scores (0.03, SD 1.31), as shown in Table 2 and Figure 2 [23].

Mean fat-free mass percentage increased by 2.3%, and mean fat mass percentage decreased by 2.5% between 36 weeks PMA and 2 years CA, as shown in Table 3.

### 3.1. Conditional Growth for Weight, Length and Head Circumference

**Figure 3:**
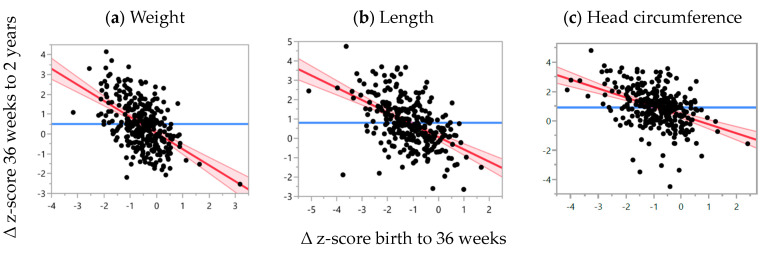
Relationship between ∆ z-score change from birth to 36 weeks PMA and ∆ weight z-score from 36 weeks PMA to 2 years CA, adjusted for birth weight z-score. (**a**) Weight: R^2^ = 0.27, ß-coefficient = −0.81 (95% CI: −0.96, −0.66), *p* < 0.0001, *n* = 318. (**b**) Length: R^2^ = 0.28, ß-coefficient = −0.64 (95% CI: −0.76, −0.51), *p* < 0.0001, *n* = 292. (**c**) HC: R^2^ = 0.18, ß-coefficient = −0.61 (95% CI: −0.76, −0.46), *p* < 0.0001, *n* = 300. Red line: line of best fit; pink area: 95% confidence interval; blue line: null model.

In the conditional growth models, for each 1 unit decrease in ∆ weight z-score from birth to 36 weeks PMA, there was, on average, a 0.81 unit (95% CI: −0.96, −0.65) increase in ∆ weight z-score from 36 weeks PMA to 2 years CA (*p* < 0.0001). This association remained statistically significant after adjusting for child ethnicity, index of deprivation, sex and small for gestational age (*p* < 0.0001), as shown in Table 4 and Figure 3. In the adjusted model, for each 1 unit decrease in weight z-score at birth, there was, on average, a 0.50 unit increase in ∆ weight z-score from 36 weeks PMA to 2 years CA (*p* < 0.0001), Table 4. Negative ∆ length z-score between birth and 36 weeks PMA was significantly associated with positive ∆ length z-score between 36 weeks PMA and 2 years CA, when adjusted for birth length z-score (*p* < 0.0001), which remained significant when adjusting for confounding factors (*p* < 0.0001), as shown in Table 4 and Figure 3. A decrease in HC z-score from birth to 36 weeks PMA was also significantly associated with increasing ∆ HC z-score between 36 weeks PMA and 2 years CA when adjusted for birth HC z-score (*p* < 0.0001) and remained significant after adjusting for confounding factors (*p* < 0.0001), as shown in Table 4 and Figure 3. Smaller birth z-scores were significantly associated with greater ∆ z-score changes between 36 weeks PMA and 2 years CA for weight (*p* < 0.0001), length (*p* < 0.0001) and HC (*p* = 0.004).

In the adjusted model, Māori children’s ∆ z-score for weight between 36 weeks PMA and 2 years CA was 0.64 (95% CI: 0.97, 0.31) (*p* = 0.0002) greater than Asian children and 0.41 (95% CI: 0.69, 0.13) (*p* = 0.005) greater than European children, as shown in Table 4. Māori children’s mean ∆ z-score for length was 0.45 (95% CI: 0.82, 0.08) (*p* = 0.02) greater than Asian children, as shown in Table 4. Adjusted ∆ z-score for weight, length and HC between 36 weeks PMA and 2 years CA was greater for Māori children than for European children, as shown in Table 4.

There were no significant differences in weight, length or HC ∆ z-score from 36 weeks PMA to 2 years CA between male and females, SGA and non-SGA and index of deprivation quintiles, as shown in Table 4.

### 3.2. Conditional Growth and Body Composition

**Figure 4:**
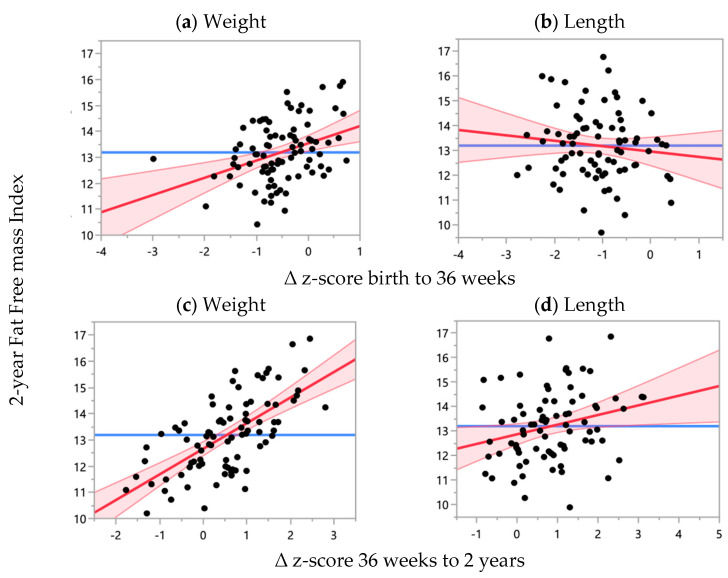
Relationship between z-score change within different time points with FFM index at 2 years CA, adjusted for ∆ z-score between birth and 36 weeks PMA, ∆ z-score between 36 weeks PMA and 2 years CA and z-score at birth for weight and length. Weight: R^2^ = 0.48, *n* = 86; length: R^2^ = 0.16, *n* = 79. (**a**) ß-coefficient = 0.66 (95% CI: 0.30, 1.03), *p* = 0.0005; (**b**) ß-coefficient = −0.22 (95% CI: −0.65, 0.22), *p* = 0.33; (**c**) ß-coefficient = 0.98 (95% CI: 0.73, 1.22), *p* < 0.0001; (**d**) ß-coefficient = 0.39 (95% CI: 0.05, 0.74), *p* = 0.03. Red line: line of best fit; pink area: 95% confidence interval; blue line: null model.

For every 1.0 unit increase in weight z-score from 36 weeks PMA to 2 years CA, there was a 0.98 Kg·m^−2^ increase in the FFM index at 2 years, when adjusted for ∆ weight z-score between birth and 36 weeks PMA and weight z-score at birth (*p* < 0.0001), as shown in Figure 4. For every 1.0 unit increase in length z-score from 36 weeks PMA to 2 years CA, there was a 0.39 Kg·m^−2^ increase in the FFM index at 2 years, when adjusted for ∆ length z-score between birth and 36 weeks PMA and length z-score at birth (*p* = 0.03), as shown in Figure 4. Positive ∆ z-score between birth and 36 weeks for weight (*p* = 0.0005), but not length, was associated with an increased FFM index at 2 years, as shown in Figure 4.

In the adjusted model, ∆ weight z-score between 36 weeks PMA and 2 years CA (*p* < 0.0001) and ∆ z-score between birth and 36 weeks (*p* = 0.01) remained significantly associated with the FFM index at 2 years CA, as shown in Table 5. For every one z-score increase in weight z-score at birth, there was a 0.55 Kg·m^−2^ increase in the FFM index at 2 years CA (*p* = 0.02), as shown in Table 5. In the adjusted model, ∆ length z-score from 36 weeks PMA to 2 years CA was no longer correlated, and ∆ length z-score between birth and 36 weeks was negatively correlated with the FFM index at 2 years CA, as shown in Table 5.

**Figure 5:**
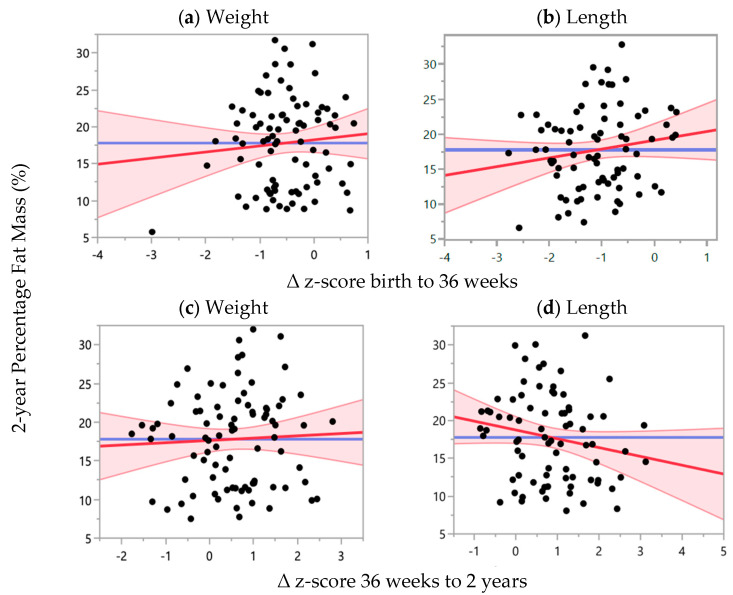
Relationship between z-score change within time points with percentage fat mass at 2 years CA, adjusted for ∆ z-score between birth and 36 weeks PMA, ∆ z-score between 36 weeks PMA and 2 years CA and z-score at birth for weight and length. Weight: R^2^ = 0.009, *n* = 86; length: R^2^ = 0.14, *n* = 79. (**a**) ß-coefficient = 0.82 (95% CI: −1.23, 2.88), *p* = 0.43; (**b**) ß-coefficient = 1.25 (95% CI: −0.55, 3.05), *p* = 0.17; (**c**). ß-coefficient =0.30 (95% CI: −1.07, 1.66), *p* = 0.67; (**d**) ß-coefficient = −1.16 (95% CI: −2.58, 0.26), *p* = 0.11. Red line: line of best fit; pink area: 95% confidence interval; blue line: null model.

There was no correlation between ∆ z-score from birth to 36 weeks PMA or between ∆ z-score from 36 weeks PMA to 2 years CA for weight or length with percent fat mass at 2 years CA, as shown in Figure 5. This remained consistent for weight after adjusting for confounding factors, as shown in Table 6. However, ∆ length z-score between birth and 36 weeks PMA (*p* = 0.048) and length z-score at birth (*p* = 0.04) were positively correlated with percentage fat mass at 2 years CA in the adjusted model. There were no significant differences in the FFM index or percentage fat mass at 2 years CA between males and females, SGA and non-SGA, different child ethnicities or index of deprivation quintiles, as shown in Table 5 and Table 6.

### 3.3. Child Eating Behaviours

The child eating behaviour with the highest mean score was enjoyment, at 3.7 (SD 1.0), and the lowest mean score was emotional over-eating, at 1.3 (SD 0.6), as shown in Table 7.

Children with BMI ≥ 90th percentile had a higher mean CEBQ score for enjoyment (4.1, SD 0.7) than children with BMI ≤ 10th percentile (3.2, SD 1.2, *p* = 0.0006) and children with BMI between 10th–90th (3.7, SD 0.9, *p* = 0.01), as shown in Table 8. Children with BMI ≥ 90th percentile also had a lower mean CEBQ score for satiety (2.2, SD 0.8) than children with BMI ≤ 10th percentile (2.8, SD 0.8, *p* = 0.009) and children with BMI between 10th–90th (2.7, SD 0.7, *p* = 0.001), as shown in Table 8.

Children with BMI ≤ 10th percentile had a higher mean CEBQ score for slowness (3.1, SD 1.0) than children with BMI between 10th–90th percentile (2.5, SD 0.8, *p* = 0.007) and children with BMI ≥ 90th percentile (2.4, SD 0.8, *p* = 0.003), as shown in Table 8. There were no significant differences in any CEBQ scores between those with a moderate/severe neurodisability, mild neurodisability or no disability.

## 4. Discussion

### 4.1. Conditional Growth

This conditional growth analysis in a large sample of ELBW babies demonstrates that slow initial growth in the NICU is associated with faster growth after discharge until 2 years CA. It also suggests that weight gain in the first 2 years is more strongly associated with fat-free mass accrual rather than with fat mass, which contradicts the existing literature [9]. While the fall in z-scores for weight, length and HC from birth to 36 weeks PMA followed by a climb in z-scores to 2 years CA in our study is consistent with reports by others [6,24], our analyses show that growth for weight, length and HC from 36 weeks PMA to 2 years CA is conditional upon growth in the NICU. Negative z-score change before NICU discharge was associated with positive z-score change between 36 weeks PMA and 2 years CA. A recent scoping review suggested that faltering growth at term CA is associated with a delay in weight, height and HC growth in childhood, although the definition for faltering growth and the populations studied differed among authors [25]. Our results suggest the opposite; slower growth in neonatal care was associated with faster growth between 36 weeks PMA and 2 years CA in this cohort, which is consistent with the patterns of growth described in a 2020 systematic review by Van de Pol and Allegaert [6]. This pattern of growth reflects catch-up growth rather than accelerated growth, as defined by Cooke et al. [5]. A longer follow-up period would be required in this cohort to determine effects on weight, height and HC growth during later childhood.

At term CA, preterm babies often have a higher percent body fat, less fat-free mass (FFM) and altered visceral fat distribution compared to healthy term-born babies [26]. In babies < 1500 g at birth, fat-free mass gains during hospital stay have been associated with improved cognitive and motor scores at 12 months CA, whilst gains in fat mass were not [27]. In the adjusted conditional growth model, z-score change for length during the NICU stay was negatively associated with the FFM index at 2 years CA. This finding may be spurious, as linear growth is often positively linked to FFM accretion [28], whilst weight is not considered an accurate reflection of fat-free mass deposition because it may be skewed by excess fat. However, this analysis also showed that an increase in weight z-score after NICU discharge was positively associated with the FFM index at 2 years CA, whilst length was not. Furthermore, neither a change in z-score for weight nor length between 36 weeks PMA and 2 years CA were correlated with percentage fat mass at 2 years CA in the conditional growth model.

Accelerated weight gain between birth and 2 years has been linked to an increased risk of childhood obesity between ages 8–11 years in a systematic review and meta-analysis (adjusted OR = 1.87; 95% CI [1.57,2.23]; *p* < 0.001) [9]. In our cohort, the correlation between change in weight z-score from 36 weeks PMA to 2 years CA and the FFM index alongside a lack of any correlation with percentage fat mass suggests that faster weight gain in the first two years after birth is mainly fat-free mass accretion rather than increased adiposity. Long-term follow-up would clarify whether the babies who had greater weight gain after 36 weeks PMA went on to develop obesity in later childhood. Ultimately, the target growth pattern for preterm babies should be one that attains the best neurodevelopmental and other long-term outcomes, but this has not yet been defined. Nevertheless, it may be important to strive for proportional growth in weight and height to obtain favourable body composition.

### 4.2. Child Eating Behaviours

Prolonged suboptimal eating behaviours can contribute to an unbalanced diet and faltering or accelerated growth. While suboptimal feeding in preterm babies is generally related to under-eating [13] linked to lower drive to eat, appetite and enjoyment of food compared to term-born children [12], the CEBQ scores in this cohort of ELBW babies were similar to CEBQ scores reported in term-born normal-weight children at 2 years [29]. However, extremely preterm-born children at age 6 have been reported to have a higher prevalence of eating problems, including oral–motor, hypersensitivity and behavioural issues, compared to term-born children [30]. The children with eating problems were also shorter, lighter and had lower mid–upper arm circumference and BMI at 6 years compared with term-born children, even after adjusting for disabilities, gestation, birthweight and feeding problems at 30 months [30]. Johnson et al. reported a higher risk of food refusal and oral motor problems in late and moderately preterm babies compared to term-born babies at 2 years, but this relationship was not significant after adjusting for prolonged nasogastric feeding, behaviour problems and delayed social competence [31]. This suggests that feeding difficulties may co-occur with other complications related to preterm birth such as neurodevelopmental or behavioural morbidities. However, we found no significant differences in any eating behaviours at 2 years between those with and without neurodevelopment disorders. Migraine et al. also reported no significant difference in drive to eat or food variety scores between children with optimal and suboptimal neurodevelopment outcomes [12].

This research identified differences in enjoyment, satiety and slowness between 2-year-olds in different BMI groups. These results were not unexpected, as the enjoyment of food is positively associated with eating responsiveness, whilst slowness and satiety are negatively associated with eating responsiveness [20]. However, it is interesting that significant differences in eating behaviours existed at 2 years and may have already affected bodyweight. Screening for eating behaviour problems may help to identify children who would benefit from intervention to support feeding practices. These results also indicate that any intervention to reduce the risk of obesity in children born at ELBW would need to be implemented before 2 years of age.

### 4.3. Strengths and Limitations

The strengths of this conditional growth analysis in ELBW babies are that we collected detailed standardised growth measurement data prospectively from birth to 2 years CA in the setting of an RCT. The follow-up rate at 2 years CA was high and the sample size was large in comparison with previously published cohort studies in ELBW babies. Participants originated from eight different sites in two countries, making the findings widely generalisable. Another strength is the analysis of eating behaviours during their first 2 years after birth in the same cohort, allowing us to determine whether any eating behaviour factors were associated with growth outcomes in the first 2 years. The main limitation is missing data. A few babies had measurements missing at some timepoints, and some questions were left blank in the questionnaire by caregivers. Body composition was measured using different methods at 36 weeks PMA and 2 years CA and was only able to be measured in a small subgroup. Follow-up beyond 2 years would help to clarify whether the babies who had greater weight gain after 36 weeks PMA went on to develop obesity in later childhood. This follow-up is currently underway.

## 5. Conclusions

This research shows growth from NICU discharge to 2 years CA is conditional upon growth in the NICU, with slower neonatal growth associated with faster early childhood growth. Positive z-score change for weight after NICU discharge is positively correlated with the fat-free mass index at 2 years CA. At age 2, children with a BMI ≥ 90th percentile have significantly different eating behaviour assessments by caregivers compared to children with a BMI ≤ 10th percentile or between the 10th–90th percentile.

Further research, specifically RCTs, is required to conclusively establish relationships between individual nutrition factors and growth outcomes in ELBW babies. Overall, the ideal growth trajectory for ELBW babies is still unclear.

## Figures and Tables

**Table 1:** Baseline demographics and characteristics of the cohort at birth.

Characteristic	All	Followed-Up at 2 Years
Neonatal factors:		
Gestational age (weeks), median (IQR)	25.7 (24.6, 26.9)	26 (24.9, 27.0)
Singleton, *n* (%)	340 (78)	262 (79)
Small for gestational age, *n* (%)	48 (11)	36 (11)
Male, *n* (%)	212 (49)	151 (46)
Maternal factors:		
Index of deprivation, median (IQR)	7 (4, 9)	7 (4, 9)
Quintile 1 (highest) *n* (%)	61 (14)	50 (15)
Quintile 2	63 (15)	51 (16)
Quintile 3	79 (18)	58 (18)
Quintile 4	92 (21)	65 (20)
Quintile 5 (lowest)	135 (31)	102 (31)
Ethnicity, *n* (%):		
Caucasian	210 (48)	163 (50)
Māori	102 (24)	77 (23)
Asian	73 (17)	57 (17)
Pacific Island	40 (9)	26 (8)
Aboriginal or Torres Strait Island	4 (1)	1 (<1)
Other	5 (1)	5 (2)

Data are presented as median (IQR) and *n* (%). Percentages may not add to 100 due to rounding. All: *n* = 434; index of deprivation, *n* = 430. Followed-up: *n* = 330; index of deprivation: *n* = 326; education, *n* = 327. Small for gestational age, birthweight < 10th percentile [17] TS, Torres Strait Islander. Data presented previously [15].

**Table 2:** Growth characteristics.

Anthropometric Measure	Birth	36 Weeks PMA	2 Years CA
Weight (g)	775 (135)	2406 (419)	12,035 (1630)
Length (cm)	32.8 (2.39)	43.6 (2.81)	86.3 (3.91)
HC (cm)	23.3 (1.55)	31.2 (1.57)	47.9 (1.84)
Weight (z-score)	0.06 (0.89)	−0.67 (0.96)	−0.14 (1.12)
Length (z-score)	−0.12 (1.04)	−1.33 (1.09)	−0.48 (1.13)
HC (z-score)	0.00 (0.90)	−0.90 (1.00)	0.03 (1.31)

Data are presented as mean (standard deviation). Weight: at birth, *n* = 434; at 36 weeks, *n* = 362; at 2 years, *n* = 321. Length: at birth, *n* = 413; at 36 weeks, *n* = 350; at 2 years, *n* = 312. HC, head circumference: at birth, *n* = 431; at 36 weeks, *n* = 356; at 2 years, *n* = 305.

**Table 3:** Body composition measurements.

Body Composition Measure	36 Weeks Post-Menstrual Age	2 Years Corrected Term Age
Fat mass (Kg)	0.62 (0.24)	2.13 (0.81)
Fat mass (%)	20.2 (4.9)	17.7 (6.1)
Fat-free mass (Kg)	2.39 (0.38)	9.88 (1.45)
Fat-free mass (%)	80.0 (4.7)	82.3 (5.9)
Fat-free mass index	10.9 (0.8)	13.2 (1.5)
Body mass index	-	16.1 (1.6)
Waist circumference (cm)	-	48.7 (5.2)
Waist–height ratio	-	0.56 (0.06)

Data are presented as mean (standard deviation); 36 weeks post-menstrual age: *n* = 20, 2 years corrected age: fat mass and fat-free mass, *n* = 87; waist circumference, *n* = 242; waist–height ratio, *n* = 238; body mass index, *n* = 311.

**Table 4:** Predictive model for ∆ z-score from 36 weeks PMA to 2 years CA for weight, length and HC adjusted for confounders.

Relationship with ∆ z-Score 36 Weeks to 2 Years		ß-Coefficient	*p*-Value
Weight	Birthweight z-score	−0.50 (−0.69, −0.32)	<0.0001
	∆ z-score birth to 36 weeks	−0.81 (−0.96, −0.65)	<0.0001
	Sex [Female]	0.03 (−0.09, 0.14)	0.65
	SGA [No]	−0.06 (−0.30, 0.19)	0.65
Length	Birth length z-score	−0.61 (−0.79, −0.44)	<0.0001
	∆ z-score birth to 36 weeks	−0.66 (−0.80, −0.52)	<0.0001
	Sex [Female]	0.07 (−0.07, 0.20)	0.34
	SGA [No]	0.17 (−0.10, 0.44)	0.21
Head circumference	Birth HC z-score	−0.32 (−0.53, −0.10)	0.004
	∆ z-score birth to 36 weeks	−0.68 (−0.84, −0.51)	<0.0001
	Sex [Female]	0.04 (−0.11, 0.19)	0.61
	SGA [No]	−0.04 (−0.33, 0.25)	0.79
∆ z-score 36 weeks to 2 years	Index of Deprivation:	Mean Difference	
Weight	Quintiles [2-1]	0.33 (−0.10, 0.76)	0.13
	Quintiles [3-2]	−0.31 (−0.72, 0.10)	0.14
	Quintiles [4-3]	0.27 (−0.10, 0.65)	0.15
	Quintiles [5-4]	−0.08 (−0.41, 0.25)	0.62
Length	Quintiles [2-1]	0.13 (−0.34, 0.60)	0.58
	Quintiles [3-2]	−0.23 (−0.71, 0.24)	0.33
	Quintiles [4-3]	0.37 (−0.07, 0.82)	0.10
	Quintiles [5-4]	−0.32 (−0.69, 0.06)	0.10
Head circumference	Quintiles [2-1]	−0.04 (−0.58, 0.49)	0.87
	Quintiles [3-2]	−0.08 (−0.59, 0.44)	0.76
	Quintiles [4-3]	−0.04 (−0.51, 0.43)	0.87
	Quintiles [5-4]	0.23 (−0.18, 0.64)	0.27
∆ z-score 36 weeks to 2 years	Child ethnicity (reference is Māori):	Mean Difference	
Weight	Pacific Islander	0.24 (−0.13, 0.62)	0.21
	Asian	−0.64 (−0.97, −0.31)	0.0002
	European	−0.41 (−0.69, −0.13)	0.005
Length	Pacific Islander	0.18 (−0.23, 0.60)	0.39
	Asian	−0.45 (−0.82, −0.08)	0.02
	European	−0.30 (−0.62, 0.01)	0.055
Head circumference	Pacific Islander	−0.40 (−0.87, 0.07)	0.09
	Asian	−0.38 (−0.79, 0.02)	0.07
	European	−0.45 (−0.79, −0.10)	0.01

Weight, length and head circumference data are presented as ß-coefficient or mean difference (95% CI). Weight R^2^ = 0.38, *n* = 254; length R^2^ = 0.33, *n* = 234; head circumference R^2^ = 0.25, *n* = 242. Adjusted for birth weight z-score, sex, SGA, New Zealand index of deprivation, and ethnicity. Small for gestational age, birthweight < 10th percentile [17]. Index of deprivation scores are on a scale of 1–5, with 1 = highest and 5 = lowest [21].

**Table 5:** Predictive model for FFM index at 2 years CA adjusted for confounders.

Relationship with FFM Index at 2 Years Corrected Age		ß-Coefficient	*p*-Value
Weight	Birth z-score	0.55 (0.09, 1.02)	0.02
	∆ z-score birth to 36 weeks	0.58 (0.13, 1.03)	0.01
	∆ z-score 36 weeks to 2 years	0.87 (0.56, 1.18)	<0.0001
	Sex [Female]	−0.06 (−0.34, 0.23)	0.69
	SGA [No]	−0.01 (−0.62, 0.60)	0.97
Length	Birth z-score	−0.25 (−0.76, 0.27)	0.34
	∆ z-score birth to 36 weeks	−0.53 (−1.01, −0.04)	0.04
	∆ z-score 36 weeks to 2 years	0.01 (−0.40, 0.42)	0.95
	Sex [Female]	−0.03 (−0.39, 0.33)	0.87
	SGA [No]	0.30 (−0.43, 1.03)	0.42
FFM Index	Index of Deprivation quintiles:	Mean Difference	
Weight	Quintiles [2-1]	0.38 (−0.70, 1.47)	0.48
	Quintiles [3-2]	0.24 (−0.71, 1.19)	0.61
	Quintiles [4-3]	−0.33 (−1.19, 0.52)	0.44
	Quintiles [5-4]	0.39 (−0.36, 1.14)	0.31
Length	Quintiles [2-1]	0.78 (−0.57, 2.13)	0.25
	Quintiles [3-2]	−0.27 (−1.47, 0.93)	0.65
	Quintiles [4-3]	0.25 (−0.81, 1.30)	0.64
	Quintiles [5-4]	0.48 (−0.42, 1.39)	0.29
FFM Index	Child ethnicity [reference is Māori]:	Mean Difference	
Weight	Pacific Islander	0.18 (−0.42, 0.78)	0.54
	Asian	−0.45 (−1.08, 0.19)	0.17
	European	0.13 (−0.34, 0.61)	0.57
Length	Pacific Islander	0.62 (−0.10, 1.34)	0.09
	Asian	−0.75 (−1.54, 0.04)	0.06
	European	0.04 (−0.55, 0.63)	0.88

Weight and length data are presented as ß-coefficient or mean difference (95% CI). Weight: R^2^ = 0.50, *n* = 76; length: R^2^ = 0.32, *n* = 69. Adjusted for ∆ z-score between birth and 36 weeks PMA, ∆ z-score between 36 weeks PMA and 2 years CA, birth z-score, sex, SGA, index of deprivation and ethnicity. Index of deprivation scores are on a scale of 1–5, with 1 = highest and 5 = lowest [21]. Small for gestational age, birthweight < 10th percentile [17].

**Table 6:** Predictive model for percentage fat mass at 2 years CA adjusted for confounders.

Relationship with Percent Fat Mass at 2 Years Corrected Age		ß-Coefficient	*p*-Value
Weight	Birth z-score	2.21 (−0.27, 4.68)	0.08
	∆ z-score birth to 36 weeks	1.84 (−0.55, 4.23)	0.13
	∆ z-score 36 weeks to 2 years	1.21 (−0.44, 2.85)	0.15
	Sex [Female]	−0.52 (−2.02, 0.99)	0.50
	SGA [No]	−1.68 (−4.92, 1.56)	0.30
Length	Birth z-score	2.23 (0.02, 4.44)	0.048
	∆ z-score birth to 36 weeks	2.24 (0.14, 4.33)	0.04
	∆ z-score 36 weeks to 2 years	−0.42 (−2.19, 1.35)	0.63
	Sex [Female]	−0.33 (−1.87, 1.21)	0.67
	SGA [No]	−2.49 (−5.64, 0.66)	0.12
Percent Fat Mass	Index of Deprivation quintiles:	Mean Difference	
Weight	Quintiles [2-1]	−3.71 (−9.45, 2.04)	0.20
	Quintiles [3-2]	−2.12 (−7.15, 2.92)	0.40
	Quintiles [4-3]	0.07 (−4.47, 4.60)	0.98
	Quintiles [5-4]	−2.33 (−6.31, 1.65)	0.25
Length	Quintiles [2-1]	−3.31 (−9.10, 2.48)	0.26
	Quintiles [3-2]	−0.51 (−5.64, 4.63)	0.84
	Quintiles [4-3]	−0.50 (−5.02, 4.02)	0.83
	Quintiles [5-4]	−3.07 (−6.97, 0.83)	0.12
Percent Fat Mass	Child ethnicity [reference is Māori]:	Mean Difference	
Weight	Pacific Islander	−0.37 (−3.54, 2.80)	0.82
	Asian	0.76 (−2.62, 4.13)	0.66
	European	−1.08 (−3.61, 1.44)	0.39
Length	Pacific Islander	0.86 (−2.23, 3.95)	0.58
	Asian	−0.93 (−4.32, 2.47)	0.59
	European	−1.10 (−3.63, 1.44)	0.39

Weight and length data are presented as ß-coefficient or mean difference (95% CI). Weight R^2^ = 0.32, *n* = 76; length R^2^ = 0.25, *n* = 69. Adjusted for ∆ z-score between birth and 36 weeks PMA, ∆ z-score 36 weeks PMA and 2 years CA, birth z-score, sex, SGA, index of deprivation and ethnicity. Index of deprivation scores are on a scale of 1–5, with 1 = highest and 5 = lowest [21]. Small for gestational age, birthweight < 10th percentile [17].

**Table 7:** Child eating behaviour scores from the CEBQ completed by parents at 2-year follow-up [20].

Child Eating Behaviour	Score
Food responsiveness	2.1 (0.9)
Emotional over-eating (OE)	1.3 (0.6)
Enjoyment	3.7 (1.0)
Desire to drink	2.5 (1.1)
Satiety	2.6 (0.8)
Slowness	2.5 (0.8)
Emotional under-eating (UE)	2.6 (0.9)
Food fussiness	2.2 (0.9)

Data are presented as mean (standard deviation). Total *n* = 264. Desire to drink: *n* = 263. Scores are on a scale of 1–5, with 1 = never and 5 = always.

**Table 8:** Child eating behaviour scores from CEBQ reported at 2-year follow-up in BMI ≤ 10th percentile, between 10–90th percentile and ≥90th percentile.

Child Eating Behaviour	BMI ≤ 10th Percentile	BMI Between 10th–90th Percentile	BMI ≥ 90th Percentile
Food responsiveness	2.1 (0.8)	2.1 (1.0)	2.4 (0.9)
Emotional OE	1.3 (0.5)	1.3 (0.6)	1.4 (0.6)
Enjoyment	3.2 (1.2) ^b^ ***	3.7 (0.9) ^c^ *	4.1 (0.7) ^b^ ***^, c^ *
Desire to drink	2.4 (0.9)	2.5 (1.08)	2.5 (1.1)
Satiety	2.8 (0.8) ^b^ **	2.67 (0.7) ^c^ **	2.2 (0.8) ^b^ **^, c^ **
Slowness	3.1 (1.0) ^a^ **^, b^ **	2.5 (0.8) ^a^ **	2.4 (0.8) ^b^ **
Emotional UE	2.7 (0.8)	2.6 (0.9)	2.5 (0.9)
Food fussiness	2.1 (1.0)	2.2 (0.9)	2.2 (0.9)

Data are presented as mean (SD), * *p* < 0.05, ** *p* < 0.01, *** *p* < 0.001. OE, over-eating. UE, under-eating. ^a^ shows a significant difference between BMI ≤ 10th percentile and BMI between 10th–90th percentile. ^b^ shows a significant difference between BMI ≤ 10th percentile and BMI ≥ 90th percentile. ^c^ shows a significant difference in group BMI between 10th–90th percentile and BMI ≥ 90th percentile. Total: ≤10th, *n* = 19; between 10th–90th percentile, *n* = 186; ≥90th, *n* = 46. Desire: ≤10th, *n* = 19; between 10th–90th percentile, *n* = 185; ≥90th, *n* = 46.

## Data Availability

Data are available on reasonable request to the Liggins Institute Data Access Committee. Data are not publicly available as the ProVIDe trial is an ongoing research project, and further analyses and publications are planned.
